# The Activation Effect of Hainantoxin-I, a Peptide Toxin from the Chinese Spider, *Ornithoctonus hainana*, on Intermediate-Conductance Ca^2+^-Activated K^+^ Channels

**DOI:** 10.3390/toxins6082568

**Published:** 2014-08-21

**Authors:** Pengfei Huang, Yiya Zhang, Xinyi Chen, Li Zhu, Dazhong Yin, Xiongzhi Zeng, Songping Liang

**Affiliations:** Key Laboratory of Protein Chemistry and Developmental Biology of the Ministry of Education, College of Life Sciences, Hunan Normal University, Changsha 410081, Hunan, China; E-Mails: huangpengfei7@163.com (P.H.); yiya-180@163.com (Y.Z.); eva8023chen@sina.com (X.C.); zlzhuli@163.com (L.Z.); dazhongyin002@126.com (D.Y.)

**Keywords:** IK channels, peptide activator, HNTX-I, selectivity, new drug design

## Abstract

Intermediate-conductance Ca^2+^-activated K^+^ (IK) channels are calcium/calmodulin-regulated voltage-independent K^+^ channels. Activation of IK currents is important in vessel and respiratory tissues, rendering the channels potential drug targets. A variety of small organic molecules have been synthesized and found to be potent activators of IK channels. However, the poor selectivity of these molecules limits their therapeutic value. Venom-derived peptides usually block their targets with high specificity. Therefore, we searched for novel peptide activators of IK channels by testing a series of toxins from spiders. Using electrophysiological experiments, we identified hainantoxin-I (HNTX-I) as an IK-channel activator. HNTX-I has little effect on voltage-gated Na^+^ and Ca^2+^ channels from rat dorsal root ganglion neurons and on the heterologous expression of voltage-gated rapidly activating delayed rectifier K^+^ channels (human ether-à-go-go-related gene; human * ERG*) in HEK293T cells. Only 35.2% ± 0.4% of the currents were activated in SK channels, and there was no effect on BK channels. We demonstrated that HNTX-I was not a phrenic nerve conduction blocker or acutely toxic. This is believed to be the first report of a peptide activator effect on IK channels. Our study suggests that the activity and selectivity of HNTX-I on IK channels make HNTX-I a promising template for designing new drugs for cardiovascular diseases.

## 1. Introduction

The intermediate-conductance Ca^2+^-activated K^+^ (IK) channels belong to the gene family of calcium/calmodulin-regulated and voltage-independent K^+^ channels (SK1/SK2/SK3 and IK) [1] and contribute to cellular functions by producing membrane hyperpolarization, thus regulating intracellular Ca^2+^ signaling. IK channels are gated solely by internal Ca^2+^, with a unit conductance of 20–85 pS. Expression of hIK1 in HEK293T cells gives rise to inwardly rectifying K^+^ currents, which are activated by submicromolar concentrations of intracellular Ca^2+^ (*EC*_50_ = 0.3 μM) [2]. From the pathophysiological perspective, disturbances of endothelial functions leading to reduced endothelium-dependent vasodilatation are present in patients with cardiovascular risk factors. Under normal physiological conditions, endothelium-dependent vasodilatation results from membrane hyperpolarization and the subsequent increase in endothelial cell (EC) Ca^2+^. The EC membrane potential is regulated by several ion channels, including cation and K^+^ channels. Of the large group of K^+^ channels, Ca^2+^-activated K^+^ channels especially play a role in controlling membrane hyperpolarization in vascular cells. Accordingly, studies by several groups have shown that organic small molecule activators of IK channels, such as NS309 (3-oxime-6,7-dichloro-1H-indole-2,3-dione) [3] and DC-EBIO (5,6-dichloro-1-ethyl-1,3-dihydro-2*H*-benzimidazole-2-one) [4], are, to some degree, efficient in halting cardiovascular disease processes in animal models [5].

There are a variety of valuable pharmacological tools available to study the contribution of specific IK channels to the mechanism of endothelium-dependent dilation in isolated vessels and to blood pressure control *in vivo*. IK channel modulators mainly comprise small organic compounds and venom-derived peptides [6]. These different classes of chemicals modulate the channel by binding to either the external or the internal face of the ion-conducting pore. Among the different ion modulators, venom-derived peptides usually block their targets with high affinity, that is, with *IC*_50_ in the micromolar or even nanomolar range, and are therefore considered as the most selective and potent ion-channel inhibitors. The disadvantage of venom-derived peptides that affect IK channels is that, to date, they are only inhibitors and not activators. Some small organic compounds also exhibit high affinity for IK channels, but at higher concentrations, they usually exert non-specific actions. A disadvantage of DC-EBIO and NS309 is that both compounds block L-type Ca^2+^ channels with an *IC*_50_ of 70 and 10 mM [7], respectively, and NS309 at micromolar concentrations also inhibits cardiac hERG K^+^ channels [3], thus raising concerns about its *in vivo* usage. However, in keeping with the uncertain or weak selectivity of some of the activators, caution is indicated in interpreting results when using higher dosages *in vitro* and *in vivo* [8]. For this reason, we screened for peptide-positive gating modulators from venom-derived peptides as alternatives to the existing small organic activators. 

**Figure 1:**
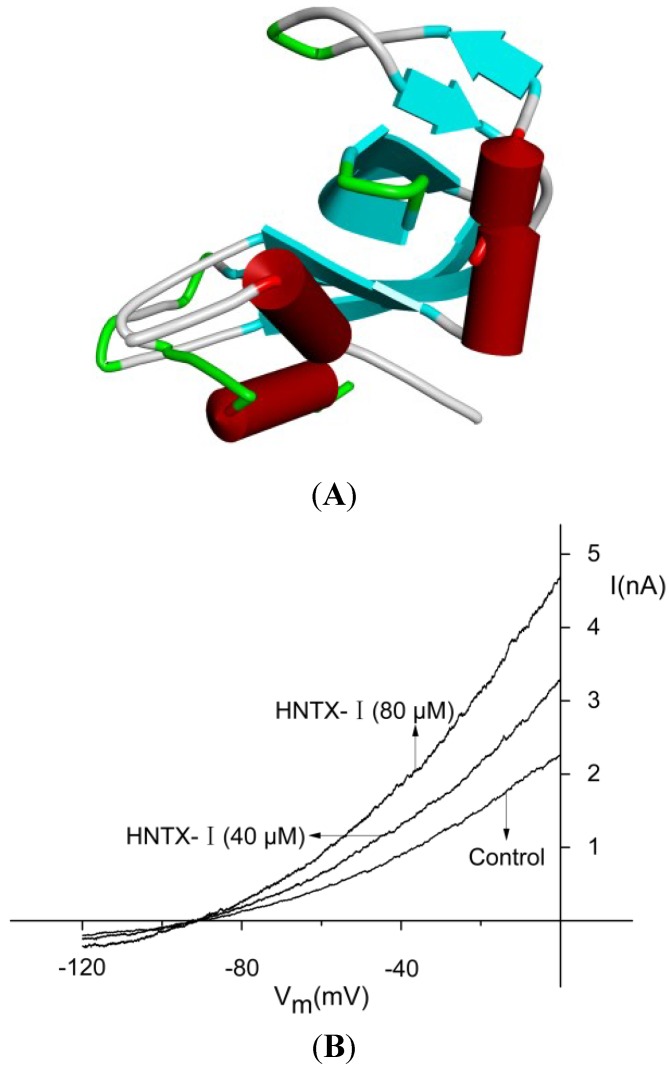
(**A**) Three-dimensional solution structure of hainantoxin-I (HNTX-I), PDB: 1N1X; (**B**) the effect of HNTX-I on whole cell currents obtained by voltage ramps applied to HEK293T cells expressing hIK1.

Spider venoms contain a variety of toxins that target ion channels and have been used as a potential source of new compounds with specific pharmacological properties. Hainantoxin-I (HNTX-I, Mu-theraphotoxin-Hhn2b, UniProtKB: D2Y1X7) is a polypeptide neurotoxin isolated from the venom of Chinese bird spider *Ornithoctonus hainana* (*O. hainana*) [9]. Composed of 33 residues and stabilized by intracellular disulfide bridges (Cys2–Cys17, Cys9–Cys22 and Cys16–Cys29), the toxin adopts a typical inhibitor cystine knot (ICK) structural motif that frequently emerges in spider toxins and conotoxins. A three-dimensional solution structure of HNTX-I has been determined using two-dimensional ^1^H-NMR spectroscopy [9] (Figure 1A, PDB: 1N1X). Our previous works demonstrated that HNTX-I shows no effect on the neuronal TTX-S VGSCs in adult rat dorsal root ganglion neurons nor does it target VGSCs in cardiac or skeletal muscles of mammals. It selectively blocks rNa_v_1.2/β1 and para/tipE channels expressed in *Xenopus laevis* oocytes. 

In the present study, IK-transfected HEK293T cells were studied in the whole-cell configuration of the patch-clamp technique. HNTX-I activated IK channels with an *EC*_50_ value of 26.3 ± 0.4 μM (*n* = 5). To test the selectivity of the compound, we screened it against a panel of other channels and revealed that voltage-gated Na^+^ channels, Ca^2+^ channels and hERG K^+^ channels were insensitive to 100 μM HNTX-I. Furthermore, a phrenic nerve conduction study and a toxicity test of mouse increase the pharmaceutical value of HNTX-I.

## 2. Results and Discussion

### 2.1. Defining the HNTX-I for hIK1 Activate

**Figure 2:**
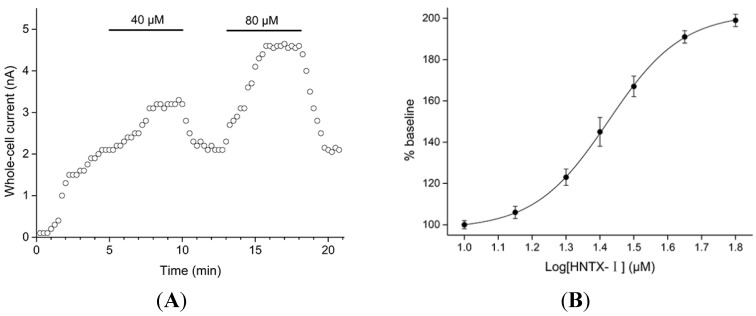
(**A**) Dose- and time-dependency of HNTX-I-induced increase in hIK1 current. The current was measured at 0 mV and plotted as a function of time (15 s between each data point) (*n* = 5). HNTX-I (40 and 80 μM) was present in the bath solution during the periods indicated by the solid bars; (**B**) the dose-response curve for HNTX-I on hIK1 current. One hundred percent denotes the baseline current level at 300 nM free Ca^2+^ concentration. The points represent the mean ± S.E. (*n* = 5).

The amino acid sequence of HNTX-I is ECKGFGKSCVPGKNECCSGYACNSRDKWCKVLL. Its experimental average molecular mass is 3,608.02 Da, and its monoisotopic molecular mass is 3605.62 Da, consistent with the calculated molecular mass for HNTX-I-amide. Hence, it was concluded that HNTX-I is amidated at the *C*-terminally. The disulfide linkages of HNTX-I were determined to be Cys2–Cys17, Cys9–Cys22 and Cys16–Cys29 (known as the 1–4, 2–5 and 3–6 disulfide patterns). The final sequence of this toxin has been confirmed by the data of disulfide bond assignment and 2D-NMR [9]. Figure 1B shows currents from a representative IK-transfected HEK293T cell elicited by voltage ramps with 0.3 μM free Ca^2+^ in the pipette solution in the presence and absence of HNTX-I, indicating that the toxin enhanced the amplitude of IK currents by 33.2% ± 0.5% and 97% ± 0.7% at the concentration of 40 and 80 μM, respectively (*n* = 5). In experiments with buffered Ca^2+^-free pipette solutions (10 mM EGTA with no added Ca^2+^), HNTX-I was not able to activate the IK channels (data not shown). The time course of an experiment on hIK1 channels is shown in Figure 2A. After 5 min of equilibration, the intracellular Ca^2+^ concentration stabilized at the new level (influenced by the buffered 0.3 μM pipette concentration). After 40 μM HNTX-I was applied, a higher current level was reached within ~3 min (*n* = 5), and upon washing, the current returned to baseline with approximately the same time characteristics. Application of 80 μM HNTX-I clearly demonstrated the dose-dependency, as well as reversible nature of this compound on hIK1 channels. HNTX-I activated the IK channels in a dose-dependent manner with an *EC*_50_ value of 26.3 ± 0.4 μM (*n* = 5, Figure 2B). 

**Figure 3:**
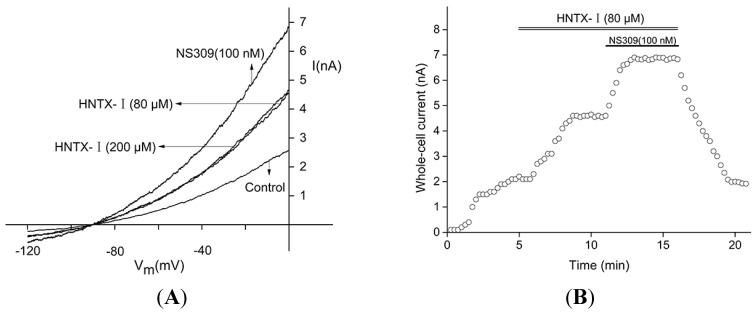
(**A**) The effect of NS309 (3-oxime-6,7-dichloro-1H-indole-2,3-dione) on whole cell currents in the presence of HNTX-I. HNTX-I activated IK channels at concentrations of 80 μM and 200 μM. NS309-modulated (100 nM NS309) IK channels co-treated with 80 and 200 μM HNTX-I; (**B**) The dose- and time-dependency of NS309 (100 nM) for the activation of hIK1 channels in the presence of HNTX-I (80 μM) (*n* = 5). The line represents the best fit to a standard Boltzmann equation with an *EC*_50_ value of 26.3 ± 0.4 μM (*n* = 5).

Defining the current shortly before the application as 100%, the *I*_max_ was 197% for 80 μM HNTX-I and did not change with a higher concentration of 200 μM. After the application of 80 μM HNTX-I, NS309 still activated the current about 1.5-fold (Figure 3A). Figure 3B shows that 100 nM NS309 activated hIK1 channels in the presence of 80 μM HNTX-I. The EC_5__0_ value for NS309 was 30 nM using the hIK1 cell line [3]. After the initial equilibration period, HNTX-I was added at 80 μM to yield a higher current within ~3 min. Then, 100 nM NS309 was applied and reached the peak in under 1 min. Thus, the potency and kinetics of NS309 were not notably influenced by HNTX-I.

In general, hydrophobic and polar residue hot spots are important binding determinants in toxins and ion channel interactions. Studies of site-directed mutagenesis in VGSCs and their toxins have demonstrated that most of the toxins act at binding sites at the outer membrane ([10,11,12,13,14,15]). In our previous work, we have shown that HNTX-I inhibits VGSCs in both vertebrates and insects. HNTX-I should therefore present a similar type of interacting surface as other toxins whose binding sites are at the outer membrane. In our previous work, we have shown that such a basic profile has also been found in HNTX-I [9]. From these data, there is a strong possibility that HNTX-I exerts extracellular binding. On the contrary, the binding pocket for the compounds of the 1-EBIO class, which penetrates cells, is located at the calmodulin interface [16,17].

### 2.2. HNTX-I Is a Highly Selective Activator of hIK1 Current

**Figure 4:**
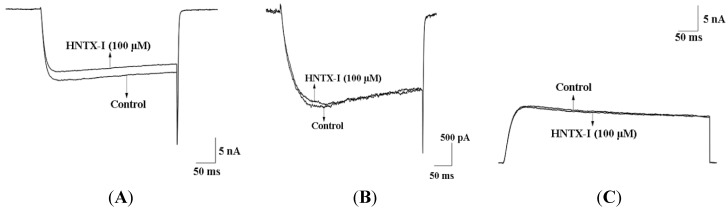
(**A**) 100 μM HNTX-I had no effect on voltage-gated L-type Ca^2+^ channels from rat dorsal root ganglia (DRG) (*n* = 5); (**B**) 100 μM HNTX-I had no effect on voltage-gated T-type Ca^2+^ channels from rat DRGs (*n* = 5); (**C**) 100 μM HNTX-I had no effect on hERG K^+^ channel in HEK293T cells (*n* = 5).

**Figure 5:**
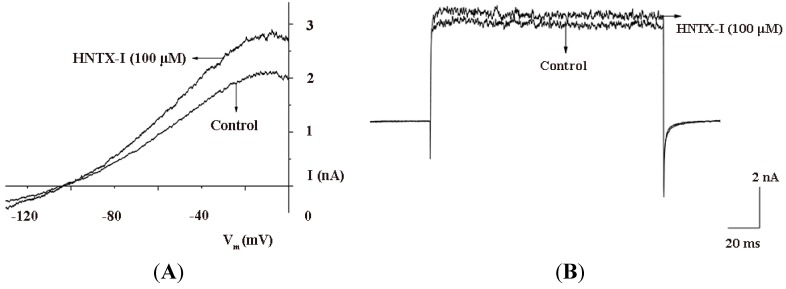
(**A**) 100 μM HNTX-I only activated about 35.2% ± 0.4% of the currents of SK1 channels (*n* = 5); (**B**) 100 μM HNTX-I had no effect on BK channels (*n* = 5).

In contrast to HNTX-I, many small organic compounds block TTX-sensitive (TTX-S) Na^+^ channels, high-threshold voltage-dependent Ca^2+^ channels, delayed-rectifier K^+^ channels and hERG K^+^channels at submicromolar concentrations [3]. HNTX-I was characterized further to test its selectivity. In our previous work [9], HNTX-I has no effect on TTX-S Na^+^ channels and tetrodotoxin-resistant (TTX-R) Na^+^ channels. Figure 4 shows the effect of externally-applied HNTX-I on L-type Ca^2+^ channels (Figure 4A), T-type Ca^2+^ channels (Figure 4B) in dorsal root ganglia (DRG) and hERG K^+^ channels (Figure 4C) in HEK293T cells. At the highest test concentrations (100 μM), HNTX-I had no effect on or blocked TTX-S Na^+^ channels, by only 10% to 20%. In summary, the voltage-gated Ca^2+^ and the voltage-gated Na^+^ and hERG K^+^ channels in HEK293T cells were insensitive to HNTX-I, which is consistent with our previous data [9]. The hERG K^+^ channel has become a primary antitarget (*i.e*., an unwanted target) in drug development, because its blockade by drugs can lead to QT prolongation. A growing list of agents with “QT liability” has been withdrawn from the market or restricted in their use [18,19]. Currently, for promising drug candidates, integration of data from hERG K^+^ channels assays with information from other pre-clinical safety screens remains essential. For this reason, HNTX-I probably offers a novel, promising treatment alternative to cardiovascular disease drugs without permanent side effects and without the risk of multiple drug interactions. Small-conductance Ca^2+^-activated K^+^ (SK) and big-conductance Ca^2+^-activated K^+^ (BK) channels are structurally similar to IK channels; therefore, we tested the SK1 and BK channels. After application of 100 μM HNTX-I, only 35.2% ± 0.4% of the currents were activated in SK channels (*n* = 5, Figure 5A), and there was no effect on BK channels (Figure 5B). Based on its high selectivity, we used HNTX-I for subsequent experiments. 

### 2.3. HNTX-I Has No Obvious Block on Phrenic Nerve Conduction in Mice

In control experiments with the preparations immersed in Tyrode’s solution, there was no significant change in the twitch responses within 4 h. HNTX-I (1 μM) did not inhibit the nerve-evoked twitch tension after a latent period of varying length. To demonstrate further that HNTX-I has little effect on phrenic nerve conduction, we examined the effects of the toxin at 10 and 100 μM concentrations, (*n* = 5), and there was still no apparent blockades in the twitch responses within 4 h. The results show that HNTX-I did not influence the neuromuscular transmission at doses of 100 μM or lower.

### 2.4. HNTX-I Is Nontoxic in an in vivo Toxicity Test

Mice injected intravenously with multiple doses of 0.5, 1.0, 1.5, 2.0 and 2.5 mg/kg HNTX-I (each doses of the toxin used in five mice) appeared clinically normal during the seven-day study. The behaviors of the HNTX-I-treated group were similar to the control mice injected with the vehicle. No aggressive behavior was directed towards another individual, and no aggressive/defensive responses were elicited. The pelage of the mice was not depilated. HNTX-I did not cause death at any concentration. We investigated the toxic effects of HNTX-I on American cockroach (*Periplaneta americana*). A large dose injection did not produce symptoms of poisoning. Collectively, data from these toxicity studies suggest that HNTX-I is not acutely toxic. HNTX-I has no obvious block on phrenic nerve conduction in mice and was safe in mice and American cockroaches. The remarkable specificity and pharmacological diversity of HNTX-I has made it a valuable source of lead molecules for drug discovery.

## 3. Experimental Section 

### 3.1. Toxins

HNTX-I from adult female *O. hainana* spider venom was collected and purified by ion-exchange chromatography and reversed-phase high performance liquid chromatography (RP-HPLC), as described in our previous work [20]. The molecular mass was determined by matrix-assisted laser desorption/ionization time-of-flight mass spectrometry (MALDI-TOF-MS). 

### 3.2. Cells

Adult Sprague-Dawley rats of both species were euthanized by CO_2_ asphyxiation and decapitated. Dorsal root ganglia (DRG) neurons were harvested from the rat spinal cord and collected in Dulbecco’s modified Eagle’s medium, then treated with protease (20 U/mL; Sigma, St. Louis, MO, USA) for 20 min followed by collagenase (0.28 U/mL; Sigma, St. Louis, MO, USA) for 40 min. Neurons were dissociated in Ham’s F12 medium supplemented with 10% horse serum, penicillin (100 U/mL), streptomycin (100 μg/mL) and L-glutamine (3.0 mM). Cells were plated on glass coverslips coated with poly-L-lysine and maintained at 37 °C in a 95% O_2_, 5% CO_2_ incubator for 24 h before electrophysiological recordings [21].

The human embryonic kidney 293T (HEK293T) cells were maintained in Dulbecco’s modified Eagle’s medium, supplemented with 100 U/mL penicillin/streptomycin, 25 mM HEPES, 10% fetal bovine serum (GIBCO-Life Technologies, Carlsbad, CA, USA) and 3 mM taurine (Sigma-Aldrich, St. Louis, MO, USA) at 37 °C and 95% O_2_, 5% CO_2_. One day before transfection, HEK293T cells were plated on 35-mm dishes (Falcon Corning-IBD, Franklin Lakes, NJ, USA) and transiently transfected with Lipofectamine 2000 Transfection Reagent (Invitrogen-Life Technologies, Carlsbad, CA, USA) according to the manufacturer’s protocol using 1 μg of either hIK1 or other channel DNA and 0.5 μg EGFP-C1 DNA. After incubation for 4–6 h, cells were replated in 35-mm culture dishes. Transfected cells were used for experiments within 1 to 2 days after transfection [22].

### 3.3. Electrophysiology

Cells were studied in the whole cell configuration of the patch-clamp technique using an EPC-10 amplifier and the Pulse program (HEKA Electronics, Mahone Bay, NS, Canada). Patch pipettes fabricated from borosilicate glass tubes using a P-97 puller (Sutter Instruments, Novato, CA, USA) were pulled to a resistance of 2.0–2.9 MΩ after heat-polishing. Membrane currents were usually filtered at 5 kHz and sampled at 20 kHz. Voltage errors were minimized using 60%–80% series-resistance compensation, and the capacitance artifact was canceled using the amplifier circuitry. Since leak currents were relatively small and can be a nonlinear function of voltage, no electronic compensation for the voltage-dependent leak current was used [23].

The holding potential in all experiments was −90 mV. For measurement of IK and SK1 currents, we used an internal pipette solution containing (in mM): 144 KCl, 10 EGTA, 7.6 CaCl_2_, 1.2 MgCl_2_ and 10 HEPES (0.3d 10 HEPES^2+^ and 1 mM free Mg^2+^), pH 7.2, 290–310 mOsm. The extracellular solution had the following composition (in mM): 140 NaCl, 4 KCl, 0.1 CaCl_2_, 3 MgCl_2_ and 10 HEPES (pH = 7.4), 290–310 mOsm [3]. IK and SK1 currents in HEK293T cells were elicited by 200-ms voltage ramps from −120 mV to 40 mV applied every 5 s, and the currents were measured at 0 mV. The BK channels were transiently expressed in HEK293T cells and studied with a pipette solution containing (in mM): 140 Mes, 160 KOH, 10 HEPES, 3.25 Ca(Mes)_2_, 3.25 CaCl_2_, pH 7.0, 290–310 mOsm; the bath solution contained (in mM): 140 Mes, 160 KOH, 2 MgCl_2_, 10 HEPES, pH 7.0, 290–310 mOsm. IK and SK1 currents in HEK293T cells were elicited by the 200-ms depolarized potential of 30 mV. The TTX-S voltage-gated Na^+^ channels on DRG neurons were studied with an internal pipette solution containing (in mM): 145 CsCl, 4 MgCl_2_, 10 HEPES, 10 EGTA, 10 glucose, 2 ATP (pH 7.2), 290–310 mOsm; and the bath solution contained (in mM): 145 NaCl, 2.5 KCl, 1.5 CaCl_2_, 1.2 MgCl_2_, 10 HEPES, 10 glucose (pH 7.4), 290–310 mOsm. Tetrodotoxin (TTX; 0.5TX) was added to the bath solution when recording TTX-R channels. The clones of hERG K^+^ channel were transiently expressed in HEK293T cells and studied with a pipette solution containing (in mM): 140 KCl, 10 HEPES, 5 EGTA, 5 ATP-Mg, and 1 MgCl_2_ (pH 7.2), 290–310 mOsm; the bath solution contained (in mM): 160 NaCl, 2.5 KCl, 2 CaCl_2_, 1 MgCl_2_, 10 HEPES, 10 glucose (pH 7.4), 290–310 mOsm. For hERG K^+^ channels recording, the currents were evoked by a 200-ms depolarized potential of 30 mV, and the reduction of the current amplitude was taken as the measure of channel blocking. Ca^2+^ channel currents from DRGs were recorded with a 200-ms depolarizing pulse of 10 mV with a pipette solution containing (in mM): 110 Cs-methane sulfonate, 14 phosphocreatine, 10 HEPES, 10 EGTA, 5 ATP-Mg (pH 7.4); and the external solution contained (in mM): 10 BaCl_2_, 125 tetraethylammonium (TEA)-Cl, 0.3 TTX and 10 HEPES (pH 7.4). For all currents elicited by voltage ramps, series resistance was not used. For calculation of *EC*_50_ values, data points were fitted using the Boltzmann equation: *y* = *A*_2_ + (*A*_1_ − *A*_2_)/1 + *e*^(*x* − *x*0)/*dx*^.

### 3.4. Blocked Studies of Phrenic Nerve Conduction 

The twitch-tension experiments were performed using mouse phrenic nerve-diaphragm preparations. Adult Kunming albino mice (18–22 g of either gender) were killed by cervical dislocation. After dissection, the preparations were placed in a Plexiglas chamber immersed in Tyrode’s solution or toxin solutions continuously bubbled with 95% O_2_, 5% CO_2_ and maintained at 30–32 °C. The electrical stimulation was applied indirectly to the phrenic nerve with a suction electrode at 0.2 Hz with pulses of 0.2 ms in duration and supramaximally. For direct stimulation, the muscle was stimulated at a frequency of 0.2 Hz (supramaximal, 2 ms, square wave). A mechanical-electric transducer made of a semiconductor strain gauge was used to transform the twitch responses into electric signals. The signals were amplified with a preamplifier (BL-420S, Chengdu, Sichuan, China) and the output from the preamplifier was instantly measured and displayed by a computer, which, in turn, creates a real-time picture on the monitor [24,25]. The time from the application of HNTX-I to complete blocking of neuromuscular transmission was defined as blocking time, and the concentrations of HNTX-I we used were 1 μM, 10 μM and 100 μM.

### 3.5. Acute in vivo Toxicity Determinations

Five mice (17–19 g) were injected intravenously with multiple 1.0-mL doses of 0.5–2.5 mg/kg HNTX-I (in mammalian Ringer solution with 1% ethanol and 2.5% BSA). Five control mice were injected with an equal volume of the vehicle. The toxicity of HNTX-I was qualitatively assayed by intra-abdominal injection into adult male cockroaches (*Periplaneta*) with body weights of 0.8–1.2 g at doses of 50–250 μg/g using 10-μL solutions (in 0.85% (*w*/*v*) normal saline). Mice and cockroaches were observed for adverse effects immediately after dosing, at 4 h after injection and daily for 7 days.

### 3.6. Statistics

Data are given as the mean ± SEM. Data sets were compared using one-way analysis of variance (ANOVA) and a paired two-tailed Student’s *t*-test if appropriate. *p*-values of <0.05 were considered statistically significant.

## 4. Conclusions 

Many studies in animals and even clinical studies have shown that decreased IK-channel activity is associated with endothelial dysfunction in cardiovascular diseases. Endothelium-dependent vasodilatation is mediated by nitric oxide (NO), prostacyclin and an endothelium-derived hyperpolarizing factor (EDHF), and it involves IK channels [5]. Endothelial dysfunction is reflected by blunted vasodilatation, and thus, opening of IK channels can increase both EDHF- and NO-mediated vasodilatation. Therefore, activator IK channels may have the potential to improve endothelial cell functions and be drug targets for the treatment of endothelial dysfunction in cardiovascular disease. Chemicals modulating IK-channel function fall into two general categories: organic small molecules and venom-derived peptides. Small organic activators, although known to exert potent pharmacological effects, have been previously described to exert non-specific actions at higher concentrations. Venom-derived peptides have proven to be useful pharmacological tools for high potency and specificity [6]. Unfortunately, to date, no peptides activating IK channels have been identified. 

In the present study, we investigated HNTX-I, which is the first selective peptide activator of IK channels. In a screening program for activity, HNTX-I activated the IK channels with an EC_50_ value of 26.3 ± 0.4 μM, which demonstrated a new positive modulator of IK channels. HNTX-I plays its full part without any distinct influence on NS309 affinity, indicating that the binding sites of these two compounds for activation may be different. In addition to this, HNTX-I had no effect on or blocked voltage-gated Na^+^, K^+^ and Ca^2+^ channels by only 10% to 20% at a concentration of 100 μM. HNTX-I had no effect on hERG K^+^ channels. 

The standard reference compounds, NS309 and DC-EBIO, have low potency and lack of selectivity, hindering their potential for clinical use. A key contributing factor is the lack of structural information about IK channels. However, the NMR solution structure of HNTX-I has been deposited in the PDB. Therefore, the 3D structure of HNTX-I provides important clues to its peptide-channel interaction. For this reason, with its great selectivity, HNTX-I is not just a promising pharmacological tool for new drug design, but it may also prove to be useful for gaining structural information about the IK channel protein itself and continue to be used widely to study IK channel gating. 
